# Therapeutic efficacy and safety of artemether-lumefantrine for the treatment of uncomplicated falciparum malaria in North-Eastern Tanzania

**DOI:** 10.1186/1475-2875-13-376

**Published:** 2014-09-20

**Authors:** Alex Shayo, Celine I Mandara, Francis Shahada, Joram Buza, Martha M Lemnge, Deus S Ishengoma

**Affiliations:** The Nelson Mandela African Institution of Science and Technology, P.O. BOX 447, Arusha, Tanzania; National Institute for Medical Research, Tanga Medical Research Centre, P.O. BOX 5004, Tanga, Tanzania

**Keywords:** Efficacy, Safety, Artemether-lumefantrine, Falciparum malaria, Tanzania

## Abstract

**Background:**

The World Health Organization recommends that regular efficacy monitoring should be undertaken by all malaria endemic countries that have deployed artemisinin combination therapy (ACT). Although ACT is still efficacious for treatment of uncomplicated malaria, artemisinin resistance has been reported in South East Asia suggesting that surveillance needs to be intensified by all malaria endemic countries. This study assessed the efficacy and safety of artemether-lumefantrine (AL) for the treatment of uncomplicated falciparum malaria in Muheza district of north-eastern Tanzania, an area where the transmission has significantly declined in recent years.

**Methods:**

Eighty eight children (aged 6 months to 10 years) with uncomplicated falciparum malaria were recruited into the study. The patients were treated with standard doses of AL and followed up for 28 days. The primary end point was parasitological cure on day 28 while the secondary end points included: improvement in haemoglobin levels and occurrence, and severity of adverse events.

**Results:**

A total of 163 febrile patients were screened, out of which 88 patients (56 under-fives and 32 aged ≥5 years) were enrolled and 79 (89.8%) completed the 28 days of follow-up. There were no cases of early treatment failure whilst 40 (78.4%) under-fives and 21(75.0%) older children had adequate clinical and parasitological response (ACPR) before PCR correction. Late clinical failure was seen in 5.6% (n = 51) and 3.6% (n = 28) of the under-fives and older children respectively; while 15.7% and 21.6% had late parasitological failure in the two groups respectively. After PCR correction, ACPR was 100% in both groups. Reported adverse events included cough (49.7%), fever (20.2%), abdominal pain (10.1%), diarrhoea (1.3%), headache (1.3%) and skin rashes (1.3%).

**Conclusion:**

This study showed that AL was safe, well-tolerated and efficacious for treatment of uncomplicated falciparum malaria. Since Muheza has historically been a hotspot of drug resistance (e.g. pyrimethamine, chloroquine, and SP), surveillance needs to be continued to detect future changes in parasite sensitivity to ACT.

## Background

Despite the current reported declining trends, malaria has remained an important public health problem and the main cause of morbidity and mortality in Tanzania and elsewhere in sub-Saharan Africa (SSA). Globally, an estimated 3.4 billion people were at risk of malaria and there were an estimated 627, 000 malaria deaths in 2012. Of the reported deaths, 90% occurred in SSA and majority involved children less than five years of age [1]. Apart from other strategies, malaria control still relies on early diagnosis and prompt treatment with efficacious drugs; and artemisinin-based combination therapy (ACT) is currently the cornerstone of malaria case management [2, 3]. Currently, ACT remains highly effective in almost all settings, as long as the partner drug in the combination is locally effective. However, the recently reported artemisinin resistance in four countries in South East Asia (Cambodia, Myanmar, Thailand and Vietnam) is a major concern for the global effort to control malaria. There is an urgent need to implement/continue with therapeutic efficacy testing (TET) in all countries which have deployed ACT as recommended by the World Health Organization (WHO) [1]. It is also crucial to intensify the surveillance of artemisinin resistance and expand containment to prevent the spread of resistance to other countries, as recommended within the WHO global plan for artemisinin resistance containment(GPARC) [4].

Following the establishment of the East Africa Network for Monitoring Antimalarial Treatment (EANMAT) in 1997, Tanzania through its National Malaria Control Programme (NMCP) has been routinely conducting TETs at eight sentinel sites located in different parts of the country [5]. The efforts by NMCP have also been complemented by trials conducted by independent researchers. The findings of these trials provided useful data to support changes of anti-malarial drug policy in Tanzania; including changes from choloroquine (CQ) to sulphadoxine-pyrimethamine (SP) in 2001 [6] and SP to artemether-lumefantrine (AL) in 2006 [7]. However, few studies have been conducted to monitor the efficacy of ACT after deployment of AL in 2006 [7] and this has been partly due to lack of funding and complacency attributed to perceived high therapeutic efficacy of ACT.

Studies conducted in Tanzania before and after introduction of ACT showed that AL has remained efficacious and safe when used for treatment of uncomplicated malaria in areas of different transmission intensity [8–11]. However, emergence of artemisinin resistance in South East Asia [12] indicates that routine monitoring needs to be urgently intensified in order to facilitate early detection of emergence and spread of tolerance/resistance to ACT in Tanzania and other malaria endemic countries. Such surveillance would provide evidence for formulating mitigation and containment strategies as recommended by WHO [4].

Furthermore, the recent decline in malaria transmission in majority of the malaria endemic countries suggests that new strategies and changes in therapeutic efficacy monitoring methods are urgently needed to ensure that monitoring of anti-malarial efficacy is routinely conducted in a sustainable manner. Such changes might include focusing on key areas in each country (e.g. areas with high levels of migrations), changing the target population (e.g. from under-fives to all age groups), reducing the cut-off density of malaria parasite and prolonging the study duration. The present study was conducted in an area where malaria has progressively and significantly declined in recent years [13] to compliment the efforts of NMCP of surveillance of anti-malarial efficacy. The study assessed the efficacy and safety of AL for treatment of uncomplicated malaria in the area which is under recent epidemiological transition from holo/hyper-endemic to low malaria transmission. It therefore, provides updates on the performance of AL after Tanzania adopted it as a first-line drug for the treatment of uncomplicated falciparum malaria [7] and in the perspectives of declining malaria transmission in the country.

## Methods

### Study site, design and target population

The study was conducted between May and August 2013 at Mkuzi Health Centre in Muheza district, north-eastern Tanzania. Mkuzi is one of the sentinel sites of the NMCP where anti-malarial TET has been routinely conducted since 1997 [5, 14]. This site and other parts of Muheza district have historically been known as a hotspot for anti-malarial drug resistance in Tanzania; including pyrimethamine [15], CQ [16, 17], amodiaquine [18] and SP [19–22]. Previous studies conducted in Muheza to test the efficacy of different ACT showed that AL had high efficacy while others such as artesunate + amodiaquine (ASAQ) and artesunate + sulphadoxine-pyrimethamine (AS-SP) had relatively low efficacy [23]. Furthermore, studies conducted at Mkuzi in 2007, showed that ASAQ had limited clinical efficacy whereby PCR uncorrected outcome of < 40% and PCR adjusted results of 80% were reported (Ishengoma et al., unpublished observation).

This study was designed as a prospective, open-label, non-randomized single-arm trial based on the WHO protocol of 2009 [24]. Unlike previous studies, the current study was conducted after the area had been reported to have a progressively declining burden of malaria [13, 25] and thus the site was considered to be under transition from high to moderate malaria transmission. The study protocol was therefore modified as recommended by WHO to include children aged six months to 10 years and parasitaemia as low as 250 asexual parasites/μl of blood [24].

### Sample size estimation

The sample size was determined based on WHO standard protocol [24] whereby treatment failure of AL was assumed to be 5%, with confidence level of 95% and a precision around the estimate of 5%. The estimated sample was 79 patients and with a 20% increase to allow for the loss to follow-up and withdrawals during the 28 days of follow-up, 88 patients were included in the study [24].

### Screening and recruitment

Children aged 6 months to 10 years who attended the outpatient department of Mkuzi Health Centre were screened, and those who met the eligibility criteria were enrolled into the study. Inclusion criteria included mono-infection of *P. falciparum* detected by microscopy, parasitaemia between 250 and 200,000 *P. falciparum* asexual forms per μl of blood and history of fever during the past 24 hours or fever at presentation (axillary temperature ≥ 37.5°C). Others were ability to swallow oral medications; ability and willingness to attend scheduled follow-up visits, informed consent provided by parent or guardian and stable residence within the catchment area throughout the study period. Patients with general danger signs or signs of severe falciparum malaria were excluded from the study. Patients with mixed or mono-infections with other Plasmodium species, presence of severe malnutrition or febrile conditions due to diseases other than malaria; and regular medication which might have interfered with anti-malarial pharmacokinetics were also excluded but received appropriate treatment according to the national guidelines.

Laboratory screening involved a finger prick to collect blood samples for detection of malaria parasites by rapid diagnostic test (RDTs) and microscopy using thick and thin smears. For patients with positive RDT results, the blood slides were stained with 10% Giemsa for 10–15 minutes and examined to detect presence of malaria parasites and the level of parasitaemia by microscopy. Patients who met the inclusion criteria were enrolled in the study and sent back to the laboratory for collection of blood samples including two more blood slides, dried blood spots (DBS) on filter papers, blood for haemoglobin determination and collection of malaria parasites for further analysis of *P. falciparum* diversity. One of the two blood slides was stained with 3% Giemsa for 30–45 min and used to determine the actual parasite density, species and presence of gametocytes. Parasitaemia was measured by counting the number of asexual parasites against 200 white blood cells (WBCs) and sexual parasites per 500 WBC in thick blood films and detection of the different parasite species was done on thin films. Parasite density per micro litre (μl) of blood was calculated by multiplying the total count by 40 for asexual and 16 for sexual parasites, assuming a putative mean count of 8,000 leucocytes/μl of blood [26]. When more than 500 parasites were identified before counting 200 leucocytes, counting was stopped and parasitaemia was calculated as stated above. A blood slide was declared negative when examination of 200 high power fields did not reveal the presence of malaria parasite.

### Treatment and follow-up

Patients enrolled in the study were treated with AL (Coartem^®^, Beijing Novartis Pharma Ltd, Beijing China for Norvatis Pharma AG, Basle, Switzerland [manufactured on August, 2012 and expiry date; July, 2014, Lot no: X1606]), a fixed combination of 20 mg of artemether and 120 mg lumefantrine in a tablet. The drugs were administered according to the recommended doses based on the weight of patient. One tablet was given to children weighing 5-14 kg; two tablets to children with 15–24 kg and three tablets to children weighing 25–35 kg. A full course of AL consisted of six doses given twice daily (8 hourly apart for day 0 and 12 hourly apart for days 1 and 2). Patients were observed for 30 minutes to ensure that they did not vomit the study drugs. When vomiting occurred, a repeat dose was given after vomiting stopped. Any patient who persistently vomited the study medication, treatment was discontinued and such patient was withdrawn from the study, and treated with parenteral quinine according to the national guidelines for management of complicated and severe malaria. Paracetamol was given to all patients with body temperature greater than or equal to 38°C. All AL doses were administered orally under direct observation of a study nurse.

Follow-up was done for 28 days with scheduled visits on days 0, 1, 2, 3, 7, 14, 21, and 28 or at any other day (unscheduled visits) when patient felt unwell. Parents/guardians were informed and encouraged to bring the children to the clinic whenever they were unwell without waiting for their scheduled visits. Patients who could not come for their scheduled visit by mid day were visited at home by a member of the study team and asked to come to the health centre. Patients who travelled to other places and could not be traced for scheduled follow-up were withdrawn from the study. During the visits, both clinical and parasitological assessments were performed; and follow-up samples (blood slides, DBS, blood for haemoglobin and parasite diversity) were also collected.

### Sample processing and parasite genotyping

Human white blood cells (WBCs) were depleted using CF11 cellulose columns [27] and parasite DNA was extracted from whole blood samples using QIAamp DNA blood midi kits (Qiagen GmbH, Hilden, Germany) according to the manufacturer’s instructions. Paired samples (day 0 and parasites collected on or after day 14) were genotyped by analysing the polymorphic loci of merozoite surface proteins 1 and 2 (MSP1 and MSP2), and glutamate rich protein (GLURP) to distinguish true recrudescent from re-infections as previously described [28, 29]. DNA extracted from blood samples collected for parasite diversity study will be sequenced at Sanger Institute, UK and the findings including the prevalence of different mutations associated with ACT resistance will be presented elsewhere.

### Outcome classification

The primary end point was parasitological cure on day 28 as per WHO protocol of 2009 [24] and secondary end points included parasitological cure on day 14, improvement in haemoglobin levels at day 28 from the day 0 baseline, and occurrence and severity of adverse events. Treatment outcomes were classified as early treatment failure (ETF), late clinical failure (LCF), late parasitological failure (LPF), and adequate clinical and parasitological response (ACPR) before and after PCR correction [30].

### Ethical considerations

Ethical clearance for this study was obtained from the National Medical Research Coordination Committee of the National Institute for Medical Research (NIMR-MRCC). Permission to conduct the study in Muheza was sought in writing from the relevant regional and district medical authorities. Oral and written informed consent was obtained from parents or guardians of all patients before they were screened for possible inclusion into the study.

### Data management and analysis

The data was entered (by double entry) into a Microsoft Access database followed by validation and cleaning. Data analysis was done using STATA for Windows, version 11 (STATA Corporation, TX-USA). Descriptive statistics as percentages, mean, median, standard deviation, and range were applied as appropriate. Treatment outcome was analysed based on per protocol method and Kaplan–Meier analysis. Patients were stratified into two groups of children aged ≥5 years and under-fives; and baseline characteristics, primary and secondary outcomes were compared between the two groups. Continuous variables such as parasite density, age and haemoglobin concentrations between the two groups were compared using t-test (for normally distributed data) or Mann–Whitney U test (a non-parametric test for non-normally distributed data). For all statistical tests, p-value was set at 0.05.

## Results

### Baseline characteristics

**Figure 1:**
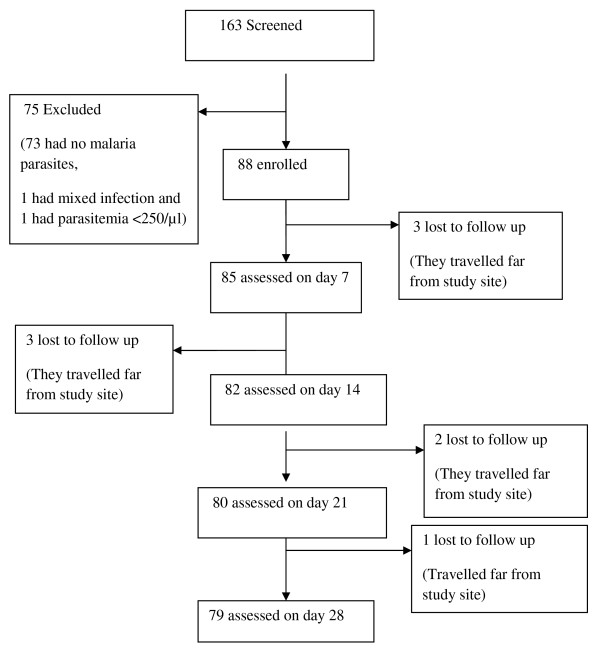
**Profile of patients screened and enrolled in the study.**

A total of 163 febrile patients were screened for eligibility to participate in the study whereby 90 (55.2%) patients had malaria parasites by microscopy and 89 (54.6%) had *P. falciparum* mono-infections. Of the *P. falciparum* mono-infected patients, one had parasite density below 250 asexual parasites per microlitre and was therefore not enrolled while 88 patients fulfilled the inclusion criteria and were enrolled into the study. During follow-up, a total of nine participants were lost to follow-up as they travelled out of the study area, leaving 79 patients for further analysis of treatment outcome (Figure 1).

Forty six (52%) of the enrolled patients were males and there were significantly more males among children aged ≥5 years compared to under-fives (Χ^2^ = 5.47, p = 0.019). The overall mean body temperature at enrolment was 38.3 ± 1.3°C while the mean haemoglobin level was 10.4 ± 1.8 g/dl. The parasite density ranged from 256 to 200,000 with a geometric mean of 18,551 asexual parasites/μl. All baseline characteristics (except sex and age) were similar in the two age groups (Table 1).

**Table 1:** **Baseline characteristics of study participants**

Variable	Overall	Under-fives	Children aged ≥5 years
Number of patient enrolled (n)	88	56	32
Age in years, mean (SD)	4.6(2.5)	3.1(1.3)	7.3(1.5)
Weight (kg), mean (SD)	16.0(5.1)	13.3(3.2)	20.7(4.4)
Sex (male)*, n (%)	46(52.3)	24(42.9)	22(68.8)
Body temperature, mean (SD)	38.3°C(1.3)	38.5°C(1.3)	38.1°C(1.3)
Geometric mean parasite density (asexual parasite/ul), 95% CI†	18,603(12,280-26,060)	19,017(12,570-28,771)	17,798(9,856-32,106)
Haemoglobin g/dL, mean (SD)	10.4(1.8)	10.2(1.9)	10.8(1.2)

SD = Standard deviation, 95% CI = 95% confidence interval.

*There were significantly more males among children aged ≥5 years compared to under-fives (Χ2 = 5.47, p = 0.019).

† Only 4 patients had parasitaemia <1000 asexual parasites/μl; parasite density ranged from 256 to 200,000 and 480 to 197,440 asexual parasites/μl in under-fives and children aged ≥5 years respectively.

### Treatment outcome

There were no cases of ETF while LCF was observed in three (5.6%) and one (3.6%) cases among under-fives and those aged ≥5 years, respectively (Table 2). Eight (15.7%) and six (21.6%) patients among under-fives and those aged ≥ 5 years, respectively, had LPF. Before PCR correction, ACPR was similar in both groups and after PCR correction, all treatment failures were shown to be due to new infections and, therefore, ACPR was 100% in both groups (Table 2).

**Table 2:** **Treatment outcome**

Outcome	PCR uncorrected	PCR corrected
**Under-fives** (n = 51)*		
Early Treatment Failure (ETF)	0 (0%)	0 (0%)
Late Clinical Failure (LCF)	3 (5.6%)***	0 (0%)
Late Parasitological Failure (LPF)	8 (15.7%)***	0 (0%)
Adequate Clinical and Parasitological Response (ACPR)	40 (78.4%)	40 (100%)
**Children aged ≥5 years** (n = 28)**		
Early Treatment Failure (ETF)	0 (0%)	0 (0%)
Late Clinical Failure (LCF)	1 (3.6%)***	0 (0%)
Late Parasitological Failure (LPF)	6 (21.6%)***	0 (0%)
Adequate Clinical and Parasitological Response (ACPR)	21 (75.0%)	21 (100%)
Overall Adequate Clinical and Parasitological Response (ACPR)	61 (77.2/%)	61 (100/%)

*5 lost to follow-up, **4 lost to follow-up.

***PCR Genotyping showed that they were new infections.

### Parasite and fever clearance

**Figure 2:**
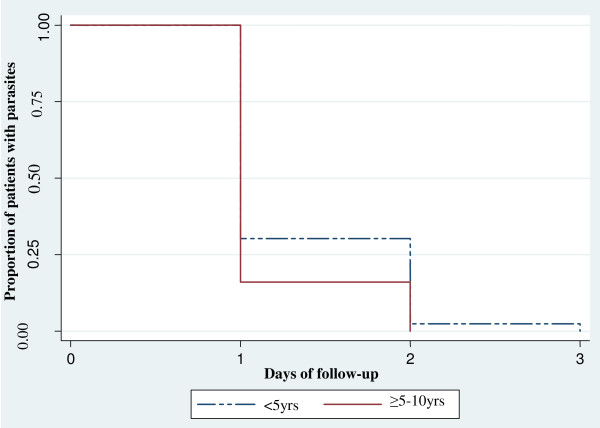
**Parasite clearance time (days) among patients treated with AL at Mkuzi Health Centre.**

**Figure 3:**
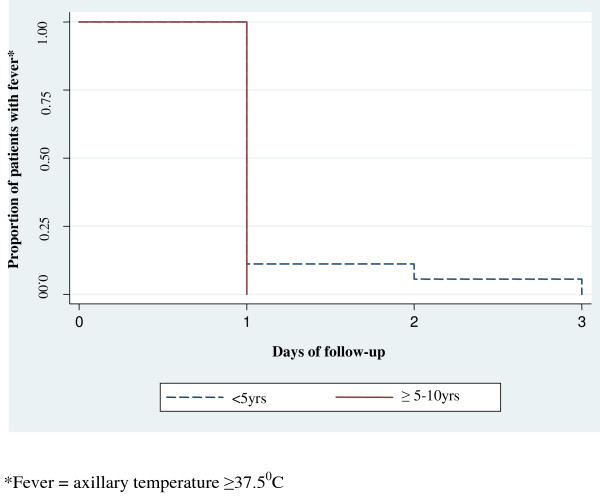
**Fever clearance time (days) among patients treated with AL at Mkuzi Health Centre.** *Fever = axillary temperature ≥37.5°C.

A total of 68 (77.3%) patients had malaria parasites on day 1 post-treatment while 17(19.5%) had parasites on day 2 and only one patient (1.4%) had parasites on day 3. Twenty three (26.1%) and 2 (2.3%) patients had fever on day 1 and 2 respectively. Two patients (2.3%) still had fever on day 3 post-treatment although the axillary temperature was low (≤37.9°C). There was no significant difference in parasite and fever clearance between under-fives and children aged ≥ 5 years (Figures 2 and 3).

### Haematological recovery

**Figure 4:**
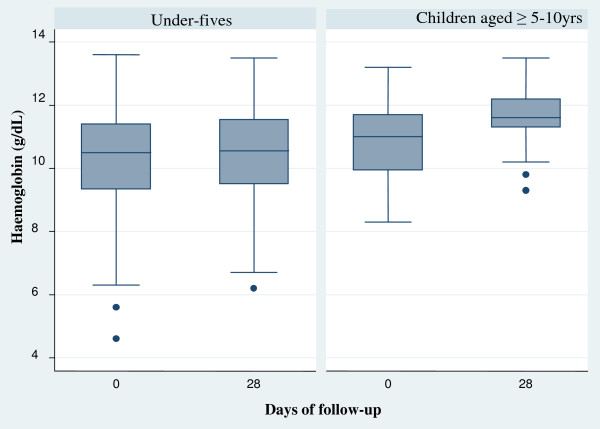
**Haemoglobin recovery in under-fives and children aged ≥5 years treated with AL.**

The mean haemoglobin at enrolment was 10.4 ± 1.8 g/dl and showed an overall progressive increase from baseline (day 0) to day 28 (day 0 *vs* day 28, p < 0.03) in both under-fives and children aged ≥5 years. The haematological recovery in children aged ≥5 years was statistically significant (p = 0.006) while Hb recovery in under-fives was not statistically significant (p = 0. 305) (Figure 4).

### Safety

Overall, adverse events (AEs) reported were cough (49.4%), fever (20.2%), abdominal pain (10.1%), diarrhoea (1.3%), headache (1.3%) and skin rashes (1.3%). However, most of the AEs occurred in under-fives compared to older children aged ≥5 years (only 6% of the under-fives had no AEs compared to about 36% of the older children). There were no serious adverse events and all the AEs were not related to the medications (Table 3).

**Table 3:** **Reported adverse events in both under-fives and children aged 5-10 years**

Adverse event	Age groups		Total
	Under-fives (n = 51)	Children aged ≥5 years (n = 28)	
Cough	28(54.9%)	11(39.3%)	39(49.4%)
Fever	13(25.5%)	3(10.7%)	16(20.2%)
Abdominal pain	5(9.8%)	3(10.7%)	8(10.1%)
Diarrhoea	1(2.0%)	0(0%)	1(1.3%)
Headache	0(0%)	1(3.6%)	1(1.3%)
Skin rashes	1(2.0%)	0(0%)	1(1.3%)
None	3(5.9%)	10(35.7%)	13(16.5%)
**Total**	**51(100%)**	**28(100%)**	**79(100%)**

## Discussion

Due to high level of resistance to common and cheap anti-malarial drugs, such as SP and CQ, Tanzania was forced to change its malaria treatment guidelines twice in a decade. The first change was done in 2001 to replace CQ (which had been used for more than three decades) with SP [6]. Because resistance to pyrimethamine which is an important partner drug in SP had been reported [21, 31] and there were indications that resistance to SP had started to emerge, the policy changes were adopted as an interim measure. Another change was, therefore, made in 2006 whereby SP was replaced with AL as the first-line for treatment of uncomplicated malaria [7]. Because of the threat of emergence and spread of artemisinin resistance in malaria endemic countries especially in Africa, in-vivo anti-malarial TET is recommended for monitoring ACT efficacy to ensure their long-term usefulness. Contrary to the recommendations by WHO [24], surveillance of anti-malarial resistance by the Tanzanian NMCP and its partners was not routinely implemented after deployment of AL in 2007. This study was, therefore, conducted to complement NMCP efforts with the aim of assessing the therapeutic efficacy and safety of AL when used for treatment of uncomplicated malaria in an area where malaria transmission has significantly and progressively declined in recent years [13, 25].

The present study showed that the standard six-dose of AL was efficacious for treatment of uncomplicated *P. falciparum* malaria with rapid fever and parasite clearance. Although the clinical efficacy was low (overall PCR uncorrected ACPR = 77.2%), PCR corrected outcome was higher in all children irrespective of age group (ACPR = 100%). These findings are consistent with other AL therapeutic efficacy studies conducted in Tanzania and elsewhere in SSA where PCR corrected ACPR was 96 - 100% [32–36]. The rates of re-infections in the present study were relatively lower compared to previous studies [37] and this could be attributed to the currently low malaria transmission in the study area. However, the transmission of malaria observed during this period was higher compared to the same period in 2011 whereby the study conducted at the same site failed to recruit sufficient number of patients (the target was 88 children but only 31 children were enrolled) over two months (Kabanywanyi *et al.*, unpublished data). In the current study, a total of 88 children were recruited in six weeks only, suggesting that local heterogeneity and micro-epidemiological factors could be the key drivers of malaria transmission in Muheza.

Rapid parasite clearance was observed whereby only one patient (1.1%) had parasitaemia on day 3 post-treatment. Studies conducted elsewhere showed similar findings whereby <1.1% the children had parasite on day three post-treatment [3, 9]. Artemether-lumefantrine achieves fast parasite clearance as a result of fast action of artemisinins. Artemisinins are rapidly absorbed and the short lived active metabolite, dihydroartemisinin quickly reduces parasite biomass leading to prompt symptomatic relief. The longer lived partner; lumefantrine, persists in the blood eliminating any remaining parasite load and thus preventing recrudescence. The observed fast parasites clearance in this study indicates that parasite tolerance/resistance has not emerged and AL is still effective in the treatment of uncomplicated falciparum malaria in the study area. However, the observed levels of re-infections suggest that lumefantrine may not be providing sufficient protection against recurrent infections during the entire period of follow-up.

The mean temperature of children recruited in the study was high (38.5°C and 38.1°C in the under-fives and children aged ≥5 years, respectively) and consistent with the reports of studies conducted elsewhere in Tanzania [10] and Ethiopia [38]. Fast fever clearance was observed in children of all age groups whereby 98% of the children had cleared the fever within 48 hours. The quick fever clearance could be explained by the fast acting property of artemisinins which clear the parasites leading to resolution of symptoms including fever [39].

The progressive haematological recovery post-treatment suggests that malaria could be the major contributing factors to the low haemoglobin levels at recruitment especially in older children. Insignificant haematological recovery irrespective of parasite clearance in the younger children suggests that other factors, such as geohelminths, may also play a key role in the chronicity of anaemia as reported in other studies [3].

In the present study, adverse events were reported in 66 (75%) of the recruited children but they were mild and not associated with the treatment. Cough was the most frequent adverse event in both age groups, which is in line with previous studies which showed that respiratory infections are common in African children with malaria [40]. Similar findings were also observed in other studies conducted in Tanzania and Ethiopia, whereby only mild adverse events were reported with cough as the most frequent symptom [3, 41]. However, since time to onset of cough was relatively evenly distributed throughout the duration of the study, cough was probably not related either to malaria or to AL treatment. Headache was only reported in older children (aged ≥ 5 years) but this could probably be attributed to increased ability to report symptoms. However, the cause of these adverse events and their possible association with AL treatment could not be established and they need to be further explored in future efficacy studies.

Due to declining malaria transmission in Muheza district and other parts of Tanzania [13, 25], the study protocol was modified to include children aged ≥5 year and low parasite density (250–200,000 asexual parasites per/μl) as recommended by WHO [24]. The current declining trend in parasite prevalence in the study area as previously reported implies that majority of the people are experiencing low exposure to infections. This may result into delayed development and overall reduction in partial immunity to malaria [42, 43]. In such settings, besides children under-five years, older children (≥5-10 years) are also at higher risk of clinical malaria [44] and treatment outcome among these two groups is not expected to be affected by the level of immunity. Furthermore, the shifting in malaria burden from young to older children as previously reported in the study area [45], was expected to lead to more older children who would be seeking treatment for malaria. In the contrary, this study recruited more under-five children compared to those aged ≥5 years and the baseline characteristics of the two groups were similar. Furthermore, the efficacy of AL was similar in the two groups.

However, there were more female children among under-fives compared to older children aged ≥5 years. The difference in the proportion of female and male in the present study may simply be related to the difference in health-seeking behaviour rather than actual difference in disease prevalence as it has been reported elsewhere [38]. Although previous studies enrolled only children below five years on the ground of low immunity to malaria, this study shows that treatment response was not affected by age. This indicates limited role of immunity in enhancing treatment response which is contrary to previous studies which showed that acquired immunity enhanced treatment response even with resistant parasites [46]. On the other hand, lowering the cut-off of parasite density to 250 asexual parasites/μl was expected to increase the number of enrolled patients. It was observed that only 4 patients had parasite density ranging from 250 – 1000 and 9 had 250 – 2000 asexual parasites per/μl suggesting that the decision to lower parasitaemia and inclusion of children aged ≥5 years in anti-malarial clinical trials needs to be further re-evaluated.

## Conclusion

The efficacy of AL for treatment of uncomplicated malaria was still high in the study area despite higher rates of re-infection. Thus, despite the reported cases of late clinical failure, these findings showed that AL was safe, well tolerated and effective for treatment of uncomplicated *P. falciparum* malaria. With the recent reports of declining trends in malaria prevalence in most parts of Tanzania including north-eastern Tanzania, anti-malarial drug pressure in the community has decreased and this may possibly limit the occurrence and spread of parasite tolerance/resistance to ACT. Despite such promising reports, as the world strives to achieve malaria elimination, further studies are needed to monitor efficacy of AL and other ACT in order to provide evidence for timely review of malaria treatment policy in the country. Since Muheza has historically been a hotspot of drug resistance (e.g. pyrimethamine, chloroquine, and SP), surveillance needs to be continued at this and other sites in the country to detect future changes in parasite sensitivity to ACT.
